# Imaging B cell maturation antigen in multiple myeloma

**DOI:** 10.1172/JCI210178

**Published:** 2026-09-15

**Authors:** Yangmeihui Song, Wenyu Song, Weibo Cai

**Affiliations:** 1 Department of Nuclear Medicine, Union Hospital, Tongji Medical College, Huazhong University of Science and Technology, Wuhan, Hubei, China.; 2 Hubei Key Laboratory of Molecular Imaging, Wuhan, Hubei, China.; 3 Hubei Provincial Clinical Medical Research Center for Medical Imaging, Wuhan, Hubei, China.; 4 Departments of Radiology and Medical Physics, University of Wisconsin-Madison, Madison, Wisconsin, USA.

## Abstract

Multiple myeloma is a systemic and spatially heterogeneous cancer of plasma cells. Available methods for diagnosing and monitoring disease do not fully capture its heterogeneity. For instance, bone marrow sampling is anatomically limited and [^18^F]FDG PET/CT reflects glucose metabolism rather than a specific target. In this issue of *JCI*, Gu et al. reported a prospective phase I study of [^68^Ga]Ga-PFBC01, a nanobody tracer targeting B cell maturation antigen (BCMA). The study presents a coherent translational pathway for [^68^Ga]Ga-PFBC01 PET and demonstrates high sensitivity, associations with tissue and circulating disease measures, and clinical management impact. By shifting myeloma imaging from metabolic assessment toward target biology, [^68^Ga]Ga-PFBC01 PET may visualize whole-body disease distribution and actionable target expression, while blood-pool activity may reflect systemic antigen biology (Figure 1).

## Challenges in diagnosis/measurement of multiple myeloma

Multiple myeloma, a cancer of plasma cells, is dispersed throughout the bone marrow and extends into paramedullary or extramedullary sites. This dispersal combined with within-patient heterogeneity poses challenges to current methods of diagnosis and measurement. Bone marrow sampling provides molecular information from the sampled site but may not fully represent disease distributed elsewhere in the body. Multiple myeloma’s notable spatial genomic heterogeneity undermines the ability to assume that any single biopsy captures all clinically relevant clones (1). Serum and urine biomarkers provide a systemic evaluation but little anatomic information, and they become less informative in oligosecretory or nonsecretory disease (2).

[^18^F]FDG PET/CT partly closes this gap. Use of the [^18^F]FDG tracer visualizes metabolically active disease and has become an important component of staging, response assessment, and measurable residual disease evaluation (3). However, low expression of hexokinase-2, the catalyst for the first step of glycolysis, may produce false-negative findings on [^18^F]FDG PET, whereas inflammation, repair, fracture, and treatment-related changes may produce persistent or nonspecific [^18^F]FDG uptake (4, 5). A tracer targeting a myeloma-associated antigen could therefore add a layer of biological specificity that metabolism alone cannot provide.

## From metabolism to target biology

B cell maturation antigen (BCMA, also known as TNFRSF17) is a plasma cell survival receptor within the BAFF/APRIL network and has emerged as a central therapeutic target in multiple myeloma (6). Imaging BCMA therefore offers the possibility of visualizing not only the distribution of myeloma, but also the availability of a clinically actionable target.

In this issue of *JCI*, Gu et al. reported a prospective, single-center phase I evaluation of [^68^Ga]Ga-PFBC01, a BCMA-directed single-domain antibody tracer (7), in multiple myeloma. Fifty participants underwent [^68^Ga]Ga-PFBC01 PET/CT, and [^18^F]FDG PET/CT was performed in 40 of these participants for a head-to-head comparison. [^68^Ga]Ga-PFBC01 tracer uptake correlated with BCMA immunohistochemical expression and with the plasma cell markers CD138 and VS38c, supporting target-specific binding. In the paired tracer cohort, [^68^Ga]Ga-PFBC01 PET demonstrated higher sensitivity and higher negative predictive value than [^18^F]FDG PET. Importantly, the [^68^Ga]Ga-PFBC01 PET-derived burden measure showed correlations of varying strength with marrow plasma cell percentage, monoclonal (M) protein level (a clinical measurement that screens for abnormal plasma cells), free light chains, and soluble BCMA. The conceptual advance in Gu et al.’s study extends beyond diagnostic performance. By directly targeting BCMA, the [^68^Ga]Ga-PFBC01 tracer shifts myeloma imaging from assessment of glucose utilization toward visualization of tumor-associated target biology. This distinction may be particularly relevant in lesions with low metabolic activity and in posttreatment settings, in which residual [^18^F]FDG uptake may be difficult to distinguish from inflammation or repair.

## A coherent translational pathway

A notable strength is the continuity of the translational framework. Gu et al.’s choice of PFBC01 arose from nanobody selection and molecular characterization through cell-binding and blocking experiments, subcutaneous and orthotopic myeloma models, treatment-response imaging, nonhuman-primate pharmacokinetic assessment, and prospective clinical evaluation. This progression provides a mechanistic basis for interpreting the human imaging findings and strengthens confidence that the clinical signal reflects a biologically characterized targeting agent. The study extends this research group’s earlier first-in-human report (8), moving beyond feasibility toward diagnostic performance, automated burden assessment, response classification, and an exploratory analysis of management impact.

## From accuracy to action

An imaging modality becomes clinically consequential when it provides information that can alter patient management. In the present study, several patients underwent treatment escalation when BCMA-avid disease was detected, and deescalation when residual [^18^F]FDG uptake was not accompanied by [^68^Ga]Ga-PFBC01 uptake (7). These observations illustrate the potential clinical contributions of [^68^Ga]Ga-PFBC01 PET: identifying disease that may be underestimated by metabolic or conventional assessment and reducing concern when residual [^18^F]FDG uptake may not represent viable BCMA-expressing myeloma. This bidirectional influence is clinically relevant because an effective imaging tool should help limit both undertreatment and overtreatment.

The disease management analysis was exploratory and demonstrated that [^68^Ga]Ga-PFBC01 PET could provide information distinct from existing assessments to influence clinical management in patients with multiple myeloma. Nevertheless, it is important to differentiate influence from benefit: a change in clinical management demonstrates the former, not the latter. These observations provide the rationale for prospective outcome-based studies of PET-informed treatment decisions. Such studies should determine whether decisions, including treatment escalation in smoldering myeloma or discontinuation of maintenance therapy, improve survival, reduce treatment-related toxicity, or optimize resource use.

## Quantifying skeletal disease burden

Gu et al. introduced an automated skeletal burden index: the summed mean standardized uptake value (SUVmean) of the thoracic, spinal, and pelvic skeleton (7). This approach addresses the diffuse, patchy, and multifocal distribution of myeloma, which differs from the delineated masses typical of many solid tumors. Automated segmentation may reduce observer dependence and facilitate reproducible assessment of major skeletal compartments. The resulting index correlated with marrow plasma cell percentage, M protein, free light chains, soluble BCMA, and metabolic tumor volume, and differed across disease stage and cytogenetic risk categories. These associations suggest that regional BCMA uptake contains quantitative information about overall disease activity.

The automated skeletal burden index measures average tracer activity within predefined skeletal regions rather than directly segmented tumor volume. It may therefore capture a composite of diffuse marrow involvement, focal lesions, physiological background, blood activity, and variation in target expression. Comparison with metabolic tumor volume, total lesion glycolysis, whole-body diffusion-weighted MRI, marrow plasma cell burden, and serological biomarkers will help define its biological meaning. Standardization of acquisition timing, reconstruction, reference tissues, segmentation, and response thresholds will be essential for multicenter use (5, 6).

## Two BCMA compartments in one scan

BCMA exists in both membrane-associated and soluble forms, introducing a circulating antigen component into the interpretation of [^68^Ga]Ga-PFBC01 PET. Cell-surface BCMA is cleaved by γ-secretase, releasing soluble BCMA into the circulation (9). This shedding reduces membrane target density and, at the same time, creates a circulating antigen pool that can itself bind BCMA-directed agents. In experimental systems, γ-secretase inhibition increases surface BCMA, reduces soluble BCMA, and improves the activity of BCMA-directed cellular therapy (10). More recently, high levels of soluble BCMA, high disease burden, low surface target density, and insufficient treatment exposure have been linked to resistance to BCMA-targeting T cell engagers in patients with multiple myeloma (11, 12). BCMA PET may therefore measure not only the distribution of disease, but the balance between tissue-associated target availability and a circulating antigen sink.

Against this background, the persistent blood-pool activity observed by Gu et al*.*, which correlated strongly with soluble BCMA and overall disease burden, may be the study’s distinctive finding (7). Blood-pool uptake, a measurement of the tracer activity remaining within the circulating blood at the time of imaging, may provide indirect information related to circulating BCMA-associated activity, raising the possibility that a single examination could inform both tissue-associated and circulating antigen compartments. At the same time, binding to soluble BCMA may influence tracer clearance, reduce the fraction of freely available tracer, elevate background activity, and lower lesion-to-blood contrast. Lesion uptake may map the spatial distribution of target-expressing disease, whereas blood-pool activity may provide information about systemic antigen burden. Lesion standardized uptake value (SUV) may reflect membrane BCMA density as well as perfusion, internalization, renal clearance, circulating antigen concentration, and imaging time.

This duality between membrane and soluble BCMA raises the hypothesis that lesion-to-blood measurements might serve as a target-to-sink index, integrating tissue-associated target signal with circulating antigen-associated activity. Employing multicompartment pharmacokinetic modeling to interpret the dynamic distribution between the blood pool and tissue targets could further isolate true antigen-binding kinetics from physiological clearance. Dynamic imaging, serial blood sampling, tracer-binding assays, and pharmacokinetic modeling could then establish whether this relationship reflects target availability more faithfully than static SUV measurements.

## Theranostic continuity

An important future application for [^68^Ga]Ga-PFBC01 PET concerns the management of patients considered for BCMA-directed therapy. BCMA is not simply an imaging marker. It is the therapeutic target of CAR T cell products (13, 14), bispecific antibodies (15), and antibody-drug conjugates (16) that have transformed the treatment of relapsed or refractory multiple myeloma. A quantitative whole-body map of BCMA-associated target availability could therefore connect diagnostic imaging with targeted treatment, consistent with the principle that molecular imaging is most valuable when it provides actionable information for therapy (17).

Potential applications include the pretreatment baseline assessment of spatially heterogeneous target expression, evaluation of bridging therapy efficacy, and longitudinal monitoring for antigen escape following cellular therapies. Loss or reduction of BCMA expression can emerge under therapeutic selection pressure and contribute to resistance (11). The correlation between [^68^Ga]Ga-PFBC01 uptake and tissue BCMA expression provides a biological foundation for investigating these applications, although predictive value will require confirmation in longitudinal, outcome-based studies in patients receiving BCMA-directed therapies.

## Toward clinical integration

Gu et al. have advanced myeloma imaging from nonspecific assessment of glucose metabolism toward whole-body, target-specific visualization of a molecule that is both a disease marker and a therapeutic entry point (7). [^68^Ga]Ga-PFBC01 PET is likely to be most informative when integrated with existing myeloma assessment. Bone marrow flow cytometry and sequencing can detect small clonal populations with high analytical sensitivity, but they sample a limited anatomical compartment. Imaging provides whole-body coverage but cannot detect microscopic disease below its spatial and contrast resolution (18). Whole-body MRI provides highly sensitive evaluation of marrow infiltration without target-specific information (19). Combining these methods may therefore provide a more complete assessment than any single test.

The combination of lesion-associated BCMA expression and circulating BCMA-related activity represents a conceptual advance in molecular imaging of hematologic malignancy. Gu et al.’s study integrated lesion detection, diffuse skeletal involvement, circulating antigen, and clinical decision making within one framework (7). With further validation through standardized acquisition and analysis protocols, longitudinal response assessment, formal comparison with minimal residual disease standards, and outcome-based evaluation in larger multicenter cohorts, [^68^Ga]Ga-PFBC01 PET could evolve from a lesion-detection tool into a whole-body measure of target availability and therapeutic vulnerability. This shift from metabolic imaging to whole-body assessment of target biology may enable more individualized treatment selection and more precise response evaluation in multiple myeloma.

## Conflict of interest

WC declares conflict of interest with the following corporations: Portrai, Inc., rTR Technovation Corporation, and Four Health Global Pharmaceuticals Inc.

## Funding support

This work is the result of NIH funding, in whole or in part, and is subject to the NIH Public Access Policy. Through acceptance of this federal funding, the NIH has been given a right to make the work publicly available in PubMed Central.

- The University of Wisconsin-Madison.
- National Institutes of Health (P30 CA014520).

## Figures and Tables

**Figure 1:**
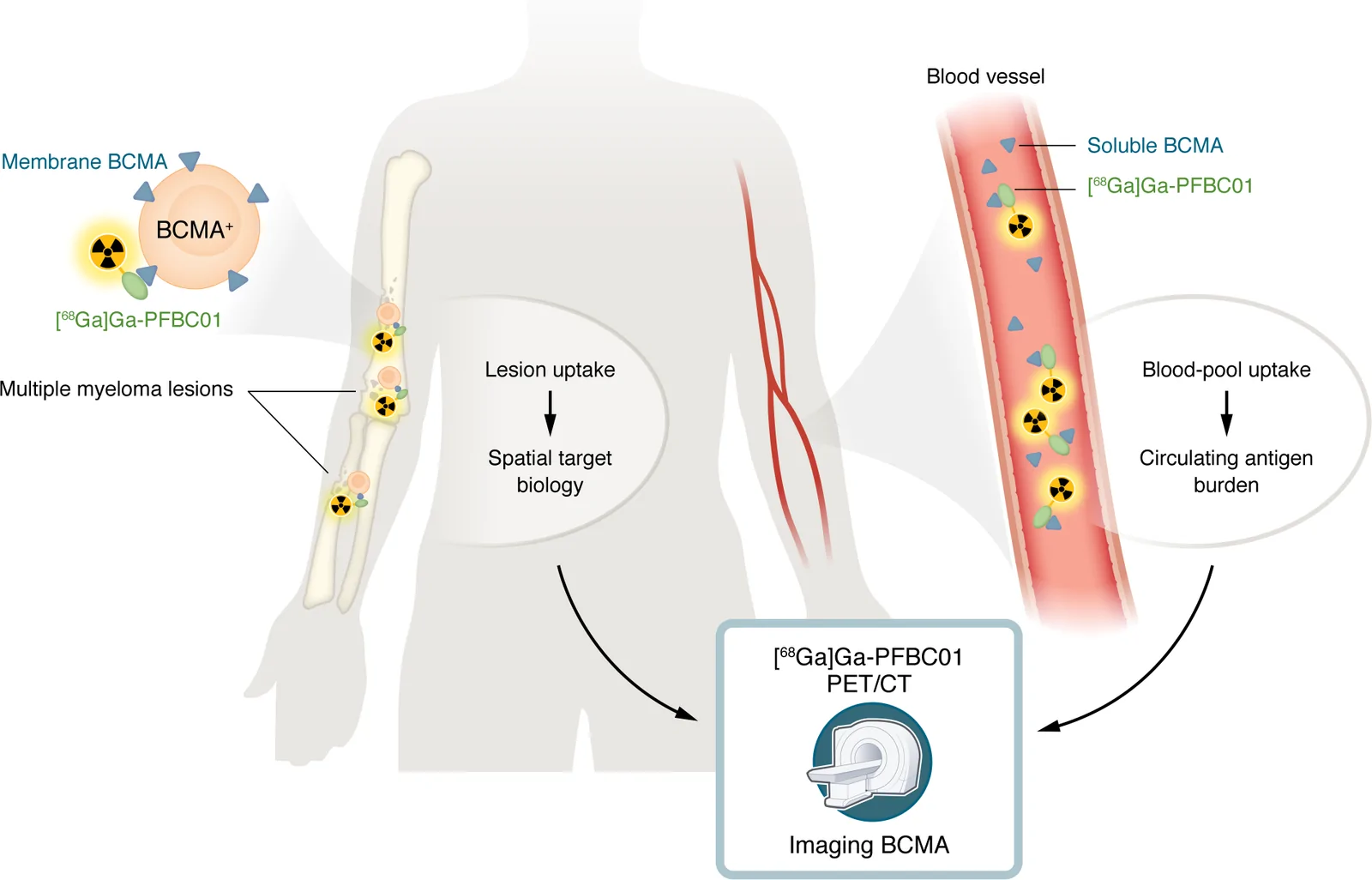
BCMA-targeted PET imaging in multiple myeloma. Gu et al. (7) showed that the [^68^Ga]Ga-PFBC01 PET tracer enabled whole-body detection of BCMA-expressing marrow and extramedullary disease and may support BCMA-directed therapy and integrated assessment of lesion and circulating BCMA burden.

## Version 1 09/15/2026

Electronic publication

## References

[1] Rasche L (2017). Spatial genomic heterogeneity in multiple myeloma revealed by multi-region sequencing. Nat Commun.

[2] Migkou M (2020). Clinical characteristics and outcomes of oligosecretory and non-secretory multiple myeloma. Ann Hematol.

[3] Cavo M (2017). Role of ^18^F-FDG PET/CT in the diagnosis and management of multiple myeloma and other plasma cell disorders: a consensus statement by the International Myeloma Working Group. Lancet Oncol.

[4] Rasche L (2017). Low expression of hexokinase-2 is associated with false-negative FDG-positron emission tomography in multiple myeloma. Blood.

[5] Zamagni E (2011). Prognostic relevance of 18-F FDG PET/CT in newly diagnosed multiple myeloma patients treated with up-front autologous transplantation. Blood.

[6] Sezer E (2025). BCMA biology and therapeutic targeting in multiple myeloma: From ligand signaling to antigen escape. Semin Hematol.

[7] Gu T (2026). A single-arm prospective phase I trial of 68Ga-PFBC01 PET/CT for multiple myeloma B cell maturation antigen imaging. J Clin Invest.

[8] Gu T (2025). B cell maturation antigen targeted PET/CT imaging in multiple myeloma: a first-in-human study. J Hematol Oncol.

[9] Laurent SA (2015). γ-Secretase directly sheds the survival receptor BCMA from plasma cells. Nat Commun.

[10] Pont MJ (2019). γ-Secretase inhibition increases efficacy of BCMA-specific chimeric antigen receptor T cells in multiple myeloma. Blood.

[11] Lee H (2023). Mechanisms of antigen escape from BCMA- or GPRC5D-targeted immunotherapies in multiple myeloma. Nat Med.

[12] Freeman CL (2025). Tumor burden quantified by soluble B cell maturation antigen and metabolic tumor volume determines myeloma CAR-T outcomes. Blood.

[13] Ravi G (2025). Phase 1 clinical trial of B cell maturation antigen (BCMA) NEX-T chimeric antigen receptor (CAR) T cell therapy CC-98633/BMS-986354 in participants with triple-class exposed multiple myeloma. Leukemia.

[14] Munshi NC (2021). Idecabtagene vicleucel in relapsed and refractory multiple myeloma. N Engl J Med.

[15] Moreau P (2022). Teclistamab in relapsed or refractory multiple myeloma. N Engl J Med.

[16] Lonial S (2020). Belantamab mafodotin for relapsed or refractory multiple myeloma (DREAMM-2): a two-arm, randomised, open-label, phase 2 study. Lancet Oncol.

[17] Weber WA (2023). What is theranostics?. J Nucl Med.

[18] Paiva B (2025). Opportunities and challenges for MRD assessment in the clinical management of multiple myeloma. Nat Rev Clin Oncol.

[19] Messiou C (2019). Guidelines for acquisition, interpretation, and reporting of whole-body MRI in myeloma: myeloma response assessment and diagnosis system (MY-RADS). Radiology.

